# Prognostic Value of Preoperative Serum Calcitonin Levels for Predicting the Recurrence of Medullary Thyroid Carcinoma

**DOI:** 10.3389/fendo.2021.749973

**Published:** 2021-10-05

**Authors:** Hyunju Park, So Young Park, Jun Park, Jun Ho Choe, Man Ki Chung, Sook-Young Woo, Joon Young Choi, Sun Wook Kim, Jae Hoon Chung, Tae Hyuk Kim

**Affiliations:** ^1^ Division of Endocrinology and Metabolism, Department of Medicine, Thyroid Center, Samsung Medical Center, Sungkyunkwan University School of Medicine, Seoul, South Korea; ^2^ Division of Endocrinology, Department of Medicine, Korea University Ansan Hospital, Ansan, South Korea; ^3^ Division of Endocrinology, Department of Medicine, Sahmyook Medical Center, Seoul, South Korea; ^4^ Division of Breast and Endocrine Surgery, Department of Surgery, Samsung Medical Center, Sungkyunkwan University School of Medicine, Seoul, South Korea; ^5^ Department of Otorhinolaryngology–Head and Neck Surgery, Samsung Medical Center, Sungkyunkwan University School of Medicine, Seoul, South Korea; ^6^ Statistics and Data Center, Samsung Medical Center, Seoul, South Korea; ^7^ Department of Nuclear Medicine, and Molecular Imaging, Samsung Medical Center, Sungkyunkwan University School of Medicine, Seoul, South Korea

**Keywords:** calcitonin, biomarker, medullary carcinoma, recurrence, prognosis

## Abstract

**Background:**

Serum calcitonin level is a useful biomarker for predicting primary tumor size, the extent of lymph node, and distant metastasis in patients with medullary thyroid carcinoma (MTC). However, the association between preoperative serum calcitonin levels and long-term oncologic outcomes has not yet been established. The aims of this study were to determine the preoperative serum calcitonin cut-off value for predicting disease recurrence and to evaluate its prognostic value.

**Methods:**

Patients with MTC (*n* = 169) who were treated at a tertiary referral hospital in Korea between 1995 and 2019 were enrolled. To determine the preoperative serum calcitonin cut-off value for predicting structural recurrence, the maximum of the standardized log-rank statistics of all possible cut-off values was used. Multivariable Cox regression analysis was used to determine prognostic factors for disease-free survival.

**Results:**

The overall disease-free survival rate was 75.7%. The preoperative serum calcitonin cut-off value that predicted structural recurrence was 309 pg/mL. Preoperative serum calcitonin levels of > 309 pg/mL were the strongest independent predictor of disease recurrence (hazard ratio (HR) 5.33, 95% confidence interval (85% CI) 1.67–16.96; *P* = 0.005). Lateral lymph node metastasis (HR 3.70, 95% CI 1.61–8.51; *P* = 0.002) and positive resection margins (HR 3.57, 95% CI 1.44–8.88; *P* = 0.006) were also significant predictors of disease recurrence.

**Conclusions:**

The preoperative serum calcitonin cut-off value is useful in clinical practice. It is also the best predictive factor for disease-free survival. Preoperative serum calcitonin levels may help determine the optimal postoperative follow-up strategy for patients with MTC.

## Introduction

Medullary thyroid carcinomas are a subtype of neuroendocrine tumors that are derived from the parafollicular cells of the thyroid gland, and secrete several hormones and peptides including calcitonin and carcinoembryonic antigen (1). Medullary thyroid carcinomas account for 3%–5% of thyroid carcinomas, with a mean age-standardized incidence of 0.19/100,000 per year (2, 3).

Tumor markers play an important role in screening for early malignancy, diagnosis, prognosis, and surveillance following curative surgery (4). Calcitonin is a tumor marker for medullary thyroid carcinoma. Measurement of serum calcitonin levels in patients with newly diagnosed, histologically confirmed medullary thyroid carcinoma is recommended by the American Thyroid Association (5). Previous studies (6–8) have shown that preoperative basal serum calcitonin levels correlate with primary tumor size, the extent of disease, and postoperative calcitonin normalization. The calcitonin doubling time has also been reported to be an independent predictor of the prognosis of medullary thyroid carcinoma. However, it is difficult to apply in practice because at least four measurements over a 2-year period are required to calculate the calcitonin doubling time (9–11). Postoperative serum calcitonin levels can also predict the recurrence of medullary thyroid carcinoma (12, 13). However, the prognostic value of preoperative basal serum calcitonin cut-off level for predicting the recurrence of medullary thyroid carcinoma has yet to be evaluated.

The aims of this study were to determine the preoperative serum calcitonin cut-off value for predicting structural recurrence and to evaluate its usefulness as a prognostic biomarker for recurrence in patients with medullary thyroid carcinoma.

## Materials and Methods

### Study Population and Ethics

The medical records of 246 patients with medullary thyroid carcinoma who were treated at the Samsung Medical Center, Seoul, Korea between 1995 and 2019 were retrospectively reviewed. Patients were excluded if preoperative serum calcitonin levels were unavailable (*n* = 71), they had inoperable advanced disease (*n* = 3), or follow-up data were missing (*n* = 3). After exclusion, 169 patients were included in the final analysis. The study design was approved by the Institutional Review Board of Samsung Medical Center (approval number: 2020-07-007). The requirement for informed consent was waived owing to the retrospective nature of the study.

### Study Outcomes and Definitions

The primary outcome of this study was to determine the preoperative serum calcitonin cut-off value for predicting structural recurrence. Structural recurrence was defined as a newly identified structural disease in the thyroid bed or neck lymph nodes or distant metastasis. A diagnosis of structural disease in the thyroid bed and/or neck lymph nodes by imaging studies was confirmed cytologically or pathologically. Distant metastases were detected by chest and/or abdominopelvic computed tomography, magnetic resonance imaging, whole-body bone scintigraphy, and 19-fluorodeoxyglucose positron emission tomography (PET) and/or were pathologically confirmed. The secondary outcomes were factors associated with disease-free survival, which was defined as the time from initial surgery to the date of first structural recurrence or last follow-up.

### Measurements of Serum Calcitonin Levels

The preoperative serum calcitonin levels were all measured by immunoradiometric assay: MEDGENIX CT-U.S.-IRMA kit (BioSource Europe S.A., Belgium) from 1995 to 2005, DSL-7700 ACTIVE IRMA kit (Diagnostic Systems Laboratories, Inc., Webster, TX) from 2005 to 2007. Since then it was replaced by current immunoradiometric assay (CT-US-IRMA, DIAsource ImmunoAssays SA, Louvain-la-Neuve, Belgium). All samples were measured in duplicate. The intra- and interassay coefficients of variation were 2.4%–3.4% and 3.6%–5.4%, respectively. The detection limit was 0.9 pg/mL.

### Statistical Analyses

Continuous variables were presented as means ± standard deviation or medians (interquartile range) and analyzed using the Student’s *t*-test or Kruskal-Wallis test, as appropriate. Categorical variables were presented as absolute numbers and percentages and analyzed using the chi-square test or Fisher’s exact test. In the derivation of the preoperative serum calcitonin cut-off value for predicting structural recurrence, running log-rank statistics were applied after removing outliers (the upper and lower 10% of patients). The preoperative serum calcitonin value that coincided with the highest log-rank statistic was chosen as the optimal cut-off value (14). A multivariable Cox proportional hazard model was constructed using backward elimination with a univariable inclusion criterion of *P* < 0.1 to assess the independent effects of covariates on disease-free survival. Statistical analyses were conducted using SPSS for Windows version 25.0 (IBM Corp., Chicago, IL, USA) and R version 4.0.1 (The R Foundation for Statistical Computing, Vienna, Austria; http://www.R-project.org/). A two-tailed *P* < 0.05 was considered statistically significant.

## Results

### Baseline Characteristics

The clinicopathological features of all patients with medullary thyroid carcinoma (*n* = 169) are summarized in **Table 1**. The mean ± standard deviation age was 49.4 ± 14.5 years; 112 patients (65.1%) were female. The median (interquartile range) follow-up was 84 (39.5–127.5) months. One hundred and sixty-seven (98.8%) and 162 patients (95.9%) underwent total thyroidectomy and central neck dissection, respectively. The primary tumor size distribution was as follows: less than or equal to 2.0 cm, 118 patients (69.8%); greater than 2.0 cm but less than or equal to 4.0 cm, 38 patients (22.5%); and greater than 4.0 cm, 13 patients (7.7%). Gross extrathyroidal extension was present in 18 patients (10.7%). Seven patients (4.1%) had positive resection margins. Central and lateral neck lymph node metastases were detected in 71 (42.0%) and 62 patients (36.7%), respectively. Ninety-four patients (55.6%) had preoperative serum calcitonin levels of > 309 pg/mL.

**Table 1:** Baseline characteristics.

Characteristics	Patients (*n* = 169)
**Age, years (mean ± SD)**	49.4 ± 14.5
**Sex, *n* (%)**	
female	112 (65.1)
male	60 (34.9)
**Tumor type, *n* (%)**	
sporadic	139 (82.2)
hereditary (MEN2A)	30 (17.8)
**Extent of surgery, *n* (%)**	
total thyroidectomy	167 (98.8)
subtotal/near total thyroidectomy	2 (1.2)
**Initial CND, *n* (%)**	
yes	162 (95.9)
no	7 (4.1)
**Tumor size, cm, *n* (%)**	
≤2.0	118 (69.8)
>2.0 and ≤4.0	38 (22.5)
>4.0	13 (7.7)
**Extrathyroidal extension, *n* (%)**	
none/micro	151 (89.3)
gross	18 (10.7)
**Resection margin, *n* (%)**	
negative	162 (95.9)
positive	7 (4.1)
**Central LNM, *n* (%)**	
no	98 (58.0)
yes	71 (42.0)
**Lateral LNM, *n* (%)**	
no	107 (63.3)
yes	62 (36.7)
**Preoperative serum calcitonin (pg/mL), *n* (%)**	
≤309	75 (44.4)
>309	94 (55.6)
**Median follow-up, month (median, IQR)**	84 (39.5-127.5)

SD, standard deviation; MEN2A, multiple endocrine neoplasia type 2A; CND, central lymph node dissection; LNM, lymph node metastasis; IQR, interquartile range.

### Clinicopathological Characteristics According to Preoperative Serum Calcitonin Levels

Clinicopathological characteristics were evaluated according to preoperative serum calcitonin levels (**Table 2**). Among 169 patients, 75 (44.4%) had preoperative serum calcitonin levels of < 309 pg/mL; the remaining 94 patients (55.6%) had preoperative serum calcitonin levels of > 309 pg/mL. Preoperative serum calcitonin levels of > 309 pg/mL were significantly associated with male sex (*P* = 0.045), a larger primary tumor size (*P* < 0.001), gross extrathyroidal extension (*P* = 0.012), and central and lateral neck lymph node metastases (*P* < 0.001).

**Table 2:** Clinicopathological characteristics according to preoperative serum calcitonin levels.

Characteristics	Calcitonin level (pg/mL)		*P*-value
	≤ 309	> 309	
**Age, years (mean ± SD)**	50.2 (12.1)	48.7 (16.2)	0.528
**Sex, *n* (%)**			
female	55 (73.3)	55 (58.5)	0.045
male	20 (26.7)	39 (41.5)
**Tumor type, *n* (%)**			
sporadic	65 (86.7)	74 (78.7)	0.179
hereditary (MEN2A)	10 (13.3)	20 (21.3)
**Extent of surgery, *n* (%)**			
total thyroidectomy	73 (97.3)	94 (100.0)	0.195
Subtotal/near total thyroidectomy	2 (2.7)	0 (0.0)
**Initial CND, *n* (%)**			
Yes	71 (94.7)	91 (96.8)	0.701
No	4 (5.3)	3 (3.2)
**Tumor size, cm, *n* (%)**			
≤2.0	70 (93.3)	48 (51.1)	<0.001
>2.0 and ≤4.0	4 (5.3)	34 (36.2)
>4	1 (1.3)	12 (12.8)
**Extrathyroidal extension, *n* (%)**			
none/micro	72 (96.0)	79 (84.0)	0.012
gross	3 (4.0)	15 (16.0)
**Resection margin, *n* (%)**			
negative	74 (98.7)	88 (93.6)	0.134
Positive	1 (1.3)	6 (6.4)
**Central LNM, *n* (%)**			
no	61 (81.3)	37 (39.4)	<0.001
yes	14 (18.7)	57 (60.6)
**Lateral LNM, *n* (%)**			
no	68 (90.7)	39 (41.5)	<0.001
yes	7 (9.3)	55 (58.5)
**Follow-up, months, median (IQR)**	96 (58–123)	80 (30.5-150.5)	0.953

SD, standard deviation; MEN2A, multiple endocrine neoplasia type 2A; CND, central lymph node dissection; LNM, lymph node metastasis; IQR, interquartile range.

### Preoperative Serum Calcitonin Cut-Off Value for Predicting Structural Recurrence

**Figure 1:**
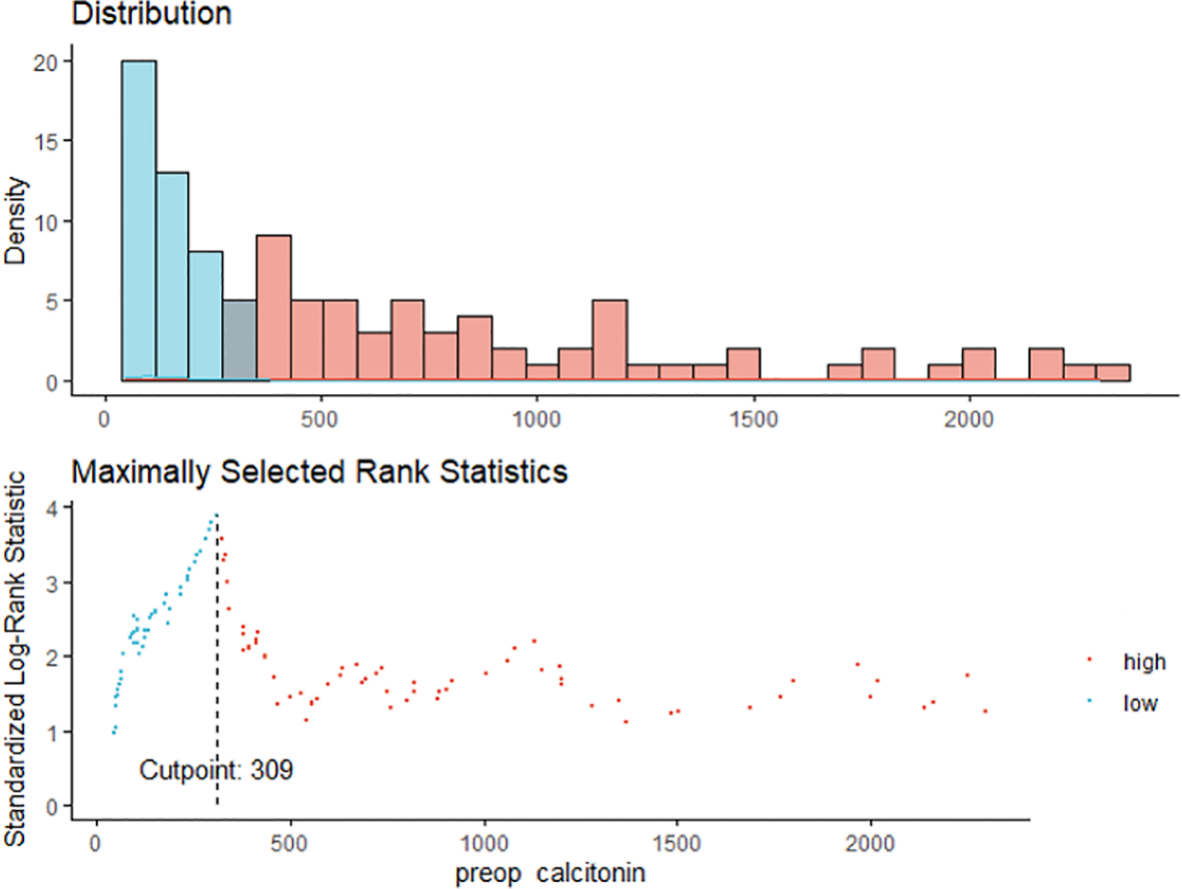
The maximum of the standardized log-rank statistics for preoperative serum calcitonin cut-off value.

**Figure 2:**
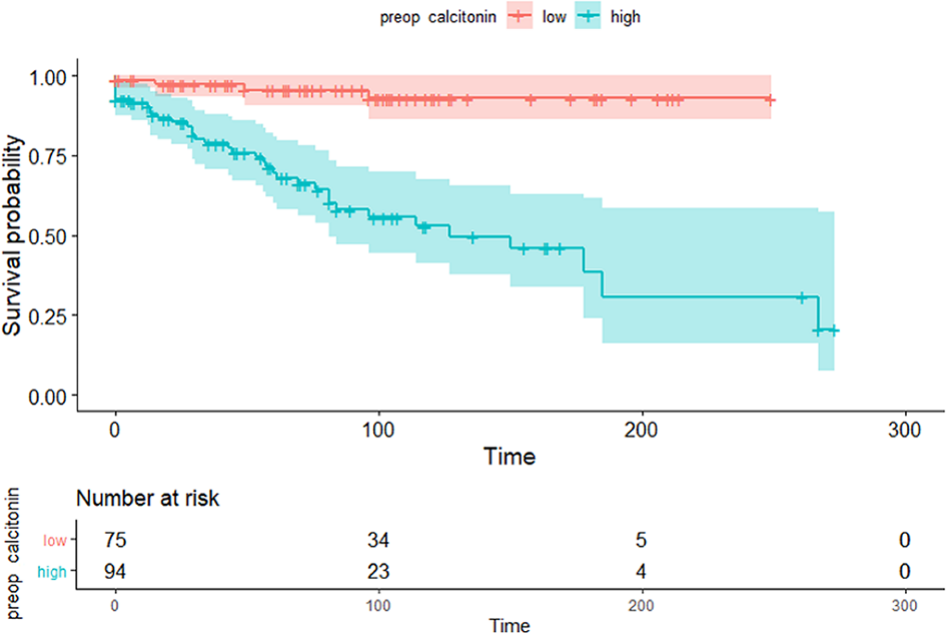
Kaplan–Meier curves of disease-free survival according to the preoperative serum calcitonin cut-off value (309 pg/mL) (*P* < 0.001).

Maximally selected log-rank statistics were applied to establish a preoperative serum calcitonin cut-off value of prognostic significance. The highest log-rank statistic coincided with a preoperative serum calcitonin level of 309 pg/mL (**Figure 1**). Kaplan–Meier curves were constructed to examine disease-free survival according to the defined preoperative serum calcitonin level (cut-off value: 309 pg/mL; **Figure 2**). The overall disease-free survival rate was 75.7%. The 10-year disease-free survival rate of patients with preoperative serum calcitonin levels of > 309 pg/mL was significantly lower than that of patients with preoperative serum calcitonin levels of < 309 pg/mL (52.9% *vs.* 92.9%, respectively; log-rank test, *P* < 0.001) (**Table 3**). Cancer-specific survival was also examined according to the defined preoperative serum calcitonin level. It also showed that preoperative serum calcitonin levels of > 309 pg/mL were associated with significantly poorer outcomes (log-rank test, *P* = 0.028; **Additional File 1: Supplementary Figure 1**).

**Table 3:** Disease-free survival and cancer-specific survival according to the preoperative serum calcitonin cut-off value of 309 pg/mL.

Calcitonin	No of patients	No of recurrences (%)	Disease-free survival (%)			
			5-year	10-year	15-year	20-year
≤309 pg/mL	75	4 (5.3)	95.5	92.9	–	–
>309 pg/mL	94	37 (39.4)	69.7	52.9	38.3	30.7
all	169	41 (24.3)	81.5	71.2	62.3	57.1
**Calcitonin**	**No of patients**	**No of deaths (%)**	Cancer-specific survival (%)*			
			**5-year**	**10-year**	**15-year**	**20-year**
≤309 pg/mL	72	0 (0.0)	–	–	–	–
>309 pg/mL	93	7 (7.5)	95.6	90.2	90.2	70.3
all	165	7 (4.2)	97.6	94.6	94.6	77.6

*Cancer-specific survival was calculated after the exclusion of four patients deaths from other causes.

### Factors Associated With Structural Recurrence

Clinical characteristics, including preoperative serum calcitonin level (≤ 309 or > 309 pg/mL), age at diagnosis, sex, extent of surgery, tumor type, primary tumor size, central and lateral neck lymph node metastasis, extrathyroidal extension, and resection margin, were analyzed as independent variables in multivariable Cox regression analysis (**Table 4**). Univariable analysis showed that preoperative serum calcitonin levels of > 309 pg/mL, a larger primary tumor size, the presence of regional lymph node metastases, gross extrathyroidal extension, and positive resection margins were associated with an increased risk of disease recurrence (*P* < 0.001). In multivariable analysis, preoperative serum calcitonin levels remained significantly associated with disease-free survival (hazard ratio: 5.33, 95% confidence interval: 1.67–16.96; *P* = 0.005). Lateral lymph node metastasis (hazard ratio: 3.70, 95% confidence interval: 1.61–8.51; *P* = 0.002) and positive resection margins (hazard ratio: 3.57, 95% confidence interval: 1.44–8.88; *P* = 0.006) were also independent predictors of disease-free survival.

**Table 4:** Multivariable analysis of disease-free survival.

Characteristics	Unadjusted		Adjusted	
	HR (95% CI)	*P* value	HR (95% CI)	*P* value
**Age, years**	0.98 (0.96-1.00)	0.104		
**Sex**				
male	1 (reference)			
female	0.61 (0.33-1.13)	0.118		
Extent of surgery*				
Subtotal/near total thyroidectomy	1 (reference)			
total thyroidectomy	non-estimable	–		
**Initial CND**				
no	1 (reference)			
yes	3.61 (0.48-27.02)	0.211		
**Tumor type**				
sporadic	1 (reference)			
hereditary	0.55 (0.21-1.40)	0.207		
**Primary tumor size, cm**		(<0.001)		(0.022)
≤2.0	1 (reference)		1 (reference)	
>2.0 and ≤4.0	1.85 (0.90-3.82)	0.095	0.53 (0.23-1.18)	0.119
>4.0	5.90 (2.71-12.84)	<0.001	1.78 (0.78-4.09)	0.177
**Central LN metastasis**				
no	1 (reference)		1 (reference)	
yes	5.30 (2.54-11.03)	<0.001	1.42 (0.52-3.86)	0.497
**Lateral LN metastasis**				
no	1 (reference)		1 (reference)	
yes	7.35 (3.47-15.59)	<0.001	3.70 (1.61-8.51)	0.002
**Extrathyroidal extension**				
none/micro	1 (reference)		1 (reference)	
Gross	4.72 (2.33-9.55)	<0.001	1.50 (0.64-3.54)	0.353
**Resection margin**				
negative	1 (reference)		1 (reference)	
positive	8.78 (3.82-20.18)	<0.001	3.57 (1.44-8.88)	0.006
**Calcitonin cut-off value, pg/mL**				
≤309	1 (reference)		1 (reference)	
>309	9.53 (3.39-26.84)	<0.001	5.33 (1.67-16.96)	0.005

*Non-estimable because all recurred patients underwent total thyroidectomy. CND, central lymph node dissection; LN, lymph node; HR, hazard ratio; 95% CI, 95% confidential interval.

## Discussion

Herein, we examined the relationship between preoperative serum calcitonin levels and the prognosis of patients with medullary thyroid carcinoma. We defined a specific preoperative serum calcitonin cut-off value of 309 pg/mL for predicting structural recurrence. We also showed that preoperative serum calcitonin levels are an accurate predictor of clinical outcomes in patients with medullary thyroid carcinoma.

Cancer biomarkers have shown potential applications in cancer detection and management (4, 15, 16). As a biomarker for medullary thyroid carcinoma (17), the routine measurement of serum calcitonin levels for detecting medullary thyroid carcinoma in patients with thyroid nodules is controversial. However, the guidelines suggest that serum calcitonin levels should be measured whenever a preoperative diagnosis of medullary thyroid carcinoma is suspected (5, 18–20). The measurement of preoperative serum calcitonin levels is simple to perform. It has been widely used for predicting the extent of regional lymph node metastases, and helps to determine the initial extent of surgery (5, 6, 21, 22). Furthermore, previous reports (7, 8, 23–25) have shown that preoperative serum calcitonin levels are associated with primary tumor size, distant metastasis, and postoperative biochemical cure.

Predicting biochemical cure is important, because postoperative biochemical cure is associated with a favorable outcome (26). Some studies have reported that preoperative serum calcitonin levels are useful for predicting disease prognosis. Cohen et al. (23) suggested the preoperative serum calcitonin levels of < 50 pg/mL could predict postoperative calcitonin normalization. Machens et al. (7) suggested that preoperative serum calcitonin levels of > 500 pg/mL could best predict the failure to achieve biochemical cure. However, they only estimated preoperative serum calcitonin levels to predict biochemical cure. Surgical resection for disease recurrence is only considered when structural recurrence has been confirmed by imaging studies (5). Thus, the preoperative serum calcitonin cut-off value for predicting structural recurrence is more important for surgical decision-making than the cut-off value for predicting biochemical cure. Yen et al. (27) investigated the relationship between preoperative serum calcitonin levels and structural recurrence-free survival. However, only a relatively small number of patients with medullary thyroid carcinoma were enrolled, and recurrence-free survival was only evaluated according to the median preoperative serum calcitonin level of enrolled patients. In this study, we defined an optimal cut-off value for preoperative serum calcitonin levels and confirmed that it had prognostic value for structural recurrence.

Previous studies (26, 28–33) have shown that age, sex, the extent of the primary tumor, extrathyroidal extension, and postoperative gross residual disease are significantly associated with oncologic outcomes. Similar factors were identified in this study. In multivariable analysis, lateral lymph node metastasis and positive resection margins were significant prognostic factors. Preoperative serum calcitonin levels of > 309 pg/mL were a poor prognostic factor for disease-free survival, and this association was maintained after adjustment for conventional risk factors for the recurrence of medullary thyroid carcinoma. The hazard ratio for a cut-off value of 309 pg/mL as a predictor for disease recurrence was 5.33, which was higher than those for conventional risk factors. Therefore, preoperative serum calcitonin levels may be a strong predictor of disease recurrence.

We also postulated that preoperative serum calcitonin levels would play an important role in predicting cancer-specific survival. A preoperative serum calcitonin cut-off value of 309 pg/mL was closely correlated with cancer-specific survival. There were no cancer-specific deaths in patients with preoperative serum calcitonin levels of < 309 pg/mL. The 5-, 10-, and 20-year cancer-specific survival rates in patients with preoperative serum calcitonin levels of > 309 pg/mL were 95.6%, 90.2%, and 70.3%, respectively. However, further multivariable analysis to identify factors affecting cancer-specific survival was not performed because only a small number of patients died from medullary thyroid carcinoma.

Postoperative biochemical remission of serum calcitonin and post-operative calcitonin doubling time were known as an important factors for oncologic outcome (13, 34). Furthermore, dynamic risk stratification has been demonstrated to be clinically valuable also in MTC (35). However, when physicians only used postoperative serum calcitonin level as predictive factor, hindsight bias which is the common tendency for people to perceive past events as having been more predictable than they actually were would be occurred (36). On the other hand, measuring preoperative calcitonin level is a simple and easy way that can be used before everything happens. Since postoperative serum calcitonin has been widely used to predict clinical outcome in real-world practice, we calculate the sensitivity and specificity for structural recurrence of each of preoperative and postoperative serum calcitonin. The sensitivity for a preoperative calcitonin level of 309 pg/mL as a predictor for structural recurrence was 90.2%, and specificity was 55.5%. The sensitivity for a postoperative calcitonin level of 10 pg/mL (7) as a predictor for structural recurrence was 80.5%, and specificity was 84.3%. The sensitivity was higher in preoperative serum calcitonin level. Using the optimal cut-off value for preoperative serum calcitonin levels for preoperative risk stratification can guide the initial resection strategy, and helpful for the determining postoperative screening test frequency for local and distant metastases. Similarly, if a patient with differentiated thyroid carcinoma was classified into high risk group according to 2009 American Thyroid Association Initial Risk Stratification System, it would not be completely reassuring even if the patient was subsequently classified into excellent response when re-evaluated using dynamic risk stratification (37).

The strength of this study lies in its relatively large number of patients with medullary thyroid carcinoma who were recruited from a single tertiary hospital. The optimal cut-off value for preoperative serum calcitonin levels was determined using maximally selected log-rank statistics, which is an appropriate statistical methodology for assessment of biomarker in survival endpoints (14, 16, 38). This study has several limitations. First is its retrospective design. Second, although this study included a relatively large number of patients with medullary thyroid carcinoma, patients were recruited from a single tertiary referral hospital. Thus, the study population may be prone to selection bias. Because of the rarity of medullary thyroid carcinoma, a multicenter prospective study is needed to validate to our findings. Third, we tried to evaluate the preoperative carcinoembryonic antigen (CEA) level concurrently, however we could not get enough information about preoperative CEA level in this cohort.

In conclusion, this study defined a preoperative serum calcitonin level of 309 pg/mL as a useful threshold for predicting disease recurrence. It also has clinical implications for long-term cancer-specific survival.

## Data Availability Statement

The raw data supporting the conclusions of this article will be made available by the authors, without undue reservation.

## Ethics Statement

The study design was approved by the Institutional Review Board of Samsung Medical Center (approval number: 2020-07-007). Written informed consent for participation was not required for this study in accordance with the national legislation and the institutional requirements.

## Author Contributions 

HP, SYP, and THK conceptualized and designed the study. HP, SYP, and S-YW analyzed the data and made the figures. HP drafted the manuscript. JP, JHCho, and MKC, data curation. SWK, JHChu, and JYC acquired and interpreted the data, and revised the manuscript. THK coordinated, and critically reviewed the manuscript for important intellectual content. All authors contributed to the article and approved the submitted version.

## Funding

This research was supported by a CRP-achievement grant (OTA1810531) from Samsung Medical Center. The funding source had no role in the collection, management, analysis, and interpretation of the data; design and conduct of the study; preparation, review, and approval of the manuscript; and decision to submit the manuscript for publication.

## Conflict of Interest

The authors declare that the research was conducted in the absence of any commercial or financial relationships that could be construed as a potential conflict of interest.

## Publisher’s Note

All claims expressed in this article are solely those of the authors and do not necessarily represent those of their affiliated organizations, or those of the publisher, the editors and the reviewers. Any product that may be evaluated in this article, or claim that may be made by its manufacturer, is not guaranteed or endorsed by the publisher.
