# Supplementary material for: Prognostic Value of Preoperative Serum Calcitonin Levels for Predicting the Recurrence of Medullary Thyroid Carcinoma
Source: Front Endocrinol (Lausanne). 2021 Oct 5;12:749973. doi: 10.3389/fendo.2021.749973 (PMC8523916; doi:10.3389/fendo.2021.749973)
Supplement: Supplementary file 1 [file DataSheet_1.docx]

**Supplementary Figure 1** | Kaplan–Meier curves of cancer-specific survival according to the preoperative serum calcitonin cut-off value (309 pg/mL) (*P* = 0.028). Cancer-specific survival curves were analysed after the exclusion of four patients with deaths from other causes.

**
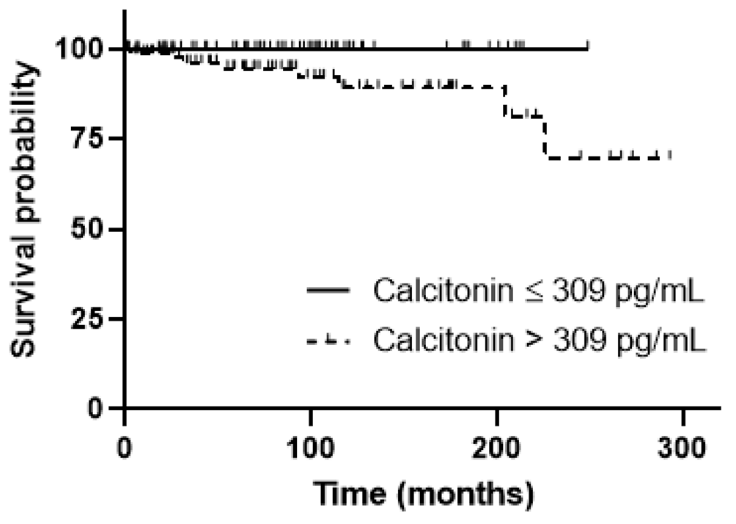
**
